# Performance of neutrophil to lymphocyte ratio for the prediction of long-term morbidity and mortality in coronary slow flow phenomenon patients presented with non-ST segment elevation acute coronary syndrome

**DOI:** 10.34172/jcvtr.2021.12

**Published:** 2021-03-01

**Authors:** Ahmet Zengin, Mehmet Karaca, Emre Aruğaslan, Ersin Yıldırım, Mehmet Baran Karataş, Yiğit Çanga, Ayşe Emre, Gülşah Tayyareci

**Affiliations:** ^1^Department of Cardiology, University of Health Scienses, Dr. Siyami Ersek Training and Research Hospital, Istanbul, Turkey; ^2^Department of Cardiology, Private Ataşehir Memorial Hospital, Istanbul, Turkey; ^3^Department of Cardiology, University of Health Scienses, Bilkent City Hospital, Ankara, Turkey; ^4^Department of Cardiology, University of Health Sciences Ümraniye Training and Research Hospital Istanbul, Turkey

**Keywords:** Neutrophil Lymphocyte Ratio, Coronary Slow Flow, Acute Coronary Syndrome

## Abstract

***Introduction:*** In this study, we aimed to determine if neutrophil to lymphocyte ratio could predict long term morbidity and mortality in patients who hospitalized for non-ST segment elevation acute coronary syndrome (NSTE-ACS) and had coronary slow flow on coronary angiography.

***Methods:*** In this observational study, 111 patients who presented with NSTE-ACS and diagnosed with coronary slow flow phenomenon on angiographic examination were included. Neutrophil to lymphocyte ratio (NLR) calculated as the ratio of the number of neutrophils to the number of lymphocytes. Patients classified into three groups according to NLR values. The term coronary slow flow phenomenon was depicted by calculating Thrombolysis in Myocardial Infarction frame count.Patients were followed up and the occurrence of recurrent angina, recurrent myocardial infarction, and long-term mortality was determined using medical records, phone calls, or face-to-face interviews. *P* values <0.05 considered to indicate statistical significance.

***Results:*** Recurrent angina and myocardial infarction occurred more frequently in the highest NLR tertile compared with middle and lowest NLR tertiles. High NLR group (NLR≥ 3.88 n=38) was significantly associated with younger age and smoking status. WBC, troponin I and CRP levels increased as the NLR tertile increased. Recurrent myocardial infarction and angina showed strong relationship with increasing NLR values. In multivariate regression analyses smoking and high NLR levels were independent predictors of recurrent myocardial infarction (HR:4.64 95%CI 0.95-22.52 P=0.04, HR: 1.48 95%CI 1.16-1.90 P<0.01 respectively) in the long term follow up.

***Conclusion:*** Our study demonstrated that high NLR values can be a valuable prognostic tool in the long term follow up of patients who presented with NSTE-ACS and diagnosed with slow flow phenomenon on coronary angiography.

## Introduction

Despite several definitions, coronary slow flow phenomenon (CSFP) was first introduced by Tambe et al. in 1972 as a different entity which is characterized by delayed distal opacification of one or more epicardial coronary arteries in the absence of stenosis.^1^ After this definition, numerous scenarios have been proposed about pathophysiology of CSFP. Microvascular dysfunction, enhanced inflammation, impaired endothelial function and vasomotor abnormalities are all attributed causes for CSFP but the exact mechanism is still unclear.^2^ The most frequent symptom is typical chest pain with exercise, but 15% of patients can be manifested as acute coronary syndromes.^3^

Inflammatory response has been well recognized in the pathogenesis of atherosclerosis. This can also play a role in the pathophysiology of CSFP.^4^ Neutrophil to lymphocyte ratio, which is known to be a marker of inflammation has been associated with adverse cardiac events. According to this, NLR is increasingly used as a marker to evaluate short and long-term prognosis in ST elevation myocardial infarction.^5,6^

There is evidence that NLR is associated with coronary slow flow phenomenon,^7^ however limited data is available about the predictive value of NLR in long term follow up of patients who present with NSTE-ACS and coronary slow phenomenon. Based on the evidence that pathophysiology and treatment of coronary slow phenomenon has not been clearly determined and the fact that most of these patients experiencing recurrent chest paint,^2^ identifying patients at risk for future cardiovascular events may have prognostic implications. We think that neutrophil to lymphocyte ratio, a simple parameter, can be used as a beneficial marker for the prediction of future adverse events in coronary slow flow phenomenon patients presented as NSTE-ACS.

## Materials and Methods

### Study population

This was a retrospectively designed observational study. After approval from the University of Health Sciences, Dr. Siyami Ersek Training and Research Hospital Ethics Committee, a total of 111 (men=75, women=36) patients diagnosed with NSTE-ACS according to their admission electrocardiography and cardiac troponin I levels and depicted coronary slow flow phenomenon on coronary angiography at the index hospitalization between the years 2011-2014 were extracted from hospitalized acute coronary syndrome patients and enrolled consecutively for the study. Systolic or diastolic heart failure, chronic renal or hepatic diseases, inflammatory or autoimmune diseases, malignancy, infection, steroid or colony stimulating factor usage have all been thought to be of exclusion criteria. Patients who had coronary plaque, atherosclerotic lesion or coronary ectasia on angiogram were also not involved for the study. Demographic features, risk factors, laboratory parameters and body mass index were recorded. Thereafter, neutrophil to lymphocyte ratio calculated manually. NSTE-ACS was defined as having typical chest pain associated with ischemic electrocardiographic changes (transient ST segment elevation, persistent or transient ST segment depression, T-wave inversion or normal ECG but not persistent ST segment elevation) and detection of cardiac Troponin-I levels above the 99^th^ percentile of the upper reference limits. Unstable angina represents the patients with negative cardiac biomarkers.^8^ A 12-derivation ECG record was obtained for all patients just after admission for the detection of MI type. The blood samples for glucose, urea, creatinine, electrolytes, C-reactive protein, lipid profile and cardiac Troponin I was obtained at the time of admission and during follow up. The blood count analyses were performed by Beckman Coulter Automated CBC Analyser (Beckman Coulter, Inc., Fullerton, CA, United States). Standard 2-dimensional echocardiographic and doppler measurements recorded by an experienced cardiologist by using the General Electric Vivid 5 (GE Health Medical, Horten, Norway).

### Coronary angiography and determination of coronary slow flow

Coronary angiography was performed by an experienced operator (>75 cases per year) using classic Judkins technique via femoral route (Siemens Axiom Artis Zee, Germany). Coronary angiographic recordings were taken at left to right oblique projections with cranial-caudal angulation at a film rate of 30 frames/seconds. Non-ionic low osmolality contrast medium (Ultravist-370 MG/ml) was used for the procedures. Coronary flow rates identified by using the Thrombolysis In Myocardial Infarction (TIMI) frame count (TFC) method^9^ This method consists calculating frame counts, which are obtained at 30 frame/seconds, until dye reaches the given distal landmarks for each coronary arteries. These landmarks defined as distal bifurcation for left anterior descending artery (LAD), first branch of the posterolateral artery for right coronary artery (RCA) and the distal bifurcation of the segment with the longest total distance for the left circumflex artery (LCx). Due to the longer course of the LAD compared with other epicardial arteries, values multiplied by constant coefficient 1.7 to standardize measurements. Published reference values are 36 ± 2.6 for LAD, 20 ± 3 for RCA and 22 ± 4 for LCx. Any TFC value greater than two standard deviation from the normal published value in the literature was accepted as coronary slow flow.

### Follow up data

Follow-up data were obtained by medical records or through patient and family member interviews. Mean duration was 35.4 ± 1.7 months. The primary outcome was the occurrence of recurrent myocardial infarction. Recurrent angina was defined as the recurrence of symptoms of angina that prompted patients to seek medical attention. Patients admitted for typical exertional or rest angina, including unstable angina, after discharge with negative cardiac biomarkers comprised the term recurrent angina. Typical angina related with characteristic electrocardiographic changes without ST segment elevation and biomarker elevation was defined as recurrent myocardial infarction when it was occurred 28 days after the index event according to the universal definition of myocardial infarction guidelines.

### Statistical analysis

Statistical analysis was performed with SPSS 17 software package. Continuous variables presented as mean ± standard deviation (SD) and categorical variables presented as numeric or percentage (%). Univariate comparisons between groups according to continuous variables distribution made by one-way ANOVA test. Chi-square test is used to compare categorical variables. Using NLR tertiles, Kaplan-Meier estimates and curves were generated, and groups were compared using Log-rank tests. Cox regression analyses were used to investigate the univariable and multivariable predictors of recurrent myocardial infarction during the study period. Forward stepwise multivariable regression models using parameters with *P* < 0.10 were created in Cox regression analyses. *P* values <0.05 considered to indicate statistical significance.

## Results

A total of 111 patients diagnosed with NSTE-ACS and found to have coronary slow flow at the index hospitalization were recruited for the study. The mean age of the patients was 50.2 ± 9.8 years and 36 (%32) were female. Based on NLR values on admission, study population divided into three distinct tertiles (1st tertile ≤ 1.87, 2nd 1.88-3.87, 3rd ≥3.88). Baseline characteristic features andthe relationship of NLR with long term follow up are shown in Table 1. Demographic features including sex, hypertension, diabetes mellitus, family history of coronary artery disease, hyperlipidemia or body mass index were similar between tertiles. Age and smoking status were significantly different in high NLR group (49.8 ± 10.4, 54.3 ± 8.6, 47.7 ± 10.9 *P* = 0.02 and %48.6, %52.7, %71 *P* = 0.04 for trend across tertiles respectively). Laboratory parameters such as hemoglobin, platelet count, creatinine or cholesterol levels were not statistically different between groups. Beside this hs-CRP (mg/dL) and WBC (10^3^/ᶙlt) levels increased as NLR tertile increased (2.1 ± 1.5 (mg/dL), 4.0 ± 1.9 (mg/dL), 6.3 ± 2.8 (mg/dL) *P* < 0.01 and 8.35 ± 1.98 (10^3^/ᶙlt), 8.44 ± 1.82 (10^3^/ᶙlt), 9.9 ± 2.7 (10^3^/ᶙlt) *P* < 0.01 respectively for trend across tertiles).

**Table 1:** Baseline characteristics and laboratory findings of the groups and relationship between NLR distribution and long-term follow-up

	**T.1 (≤1.87)** **(n=37)**	**T.2 (1.88-3.87)** **(n=36)**	**T.3 (≥3.88)** **(n=38)**	***P*** ** value**
Age, y	49.8 ± 10.4	54.3 ± 8.6	47.7 ± 10.9	0.02
Male sex (n%)	27 (%72.9)	20 (%55)	28 (%73.6)	0.17
Hypertension (n%)	12 (%32)	19 (%52.7)	15 (%39)	0.20
Diabetes mellitus (n%)	2 (%5.4)	4 (%11.1)	3 (%7.9)	0.67
Hyperlipidemia (n%)	7 (%18.9)	9 (%25)	8 (%21.0)	0.26
Smoking (n%)	18 (%48.6)	19 (%52.7)	27 (%71)	0.04
BMI, mean ± SD, kg/m2	25.3 ± 2.3	25.8 ± 2.5	25.3 ± 2.6	0.67
Family history, (n%)	12 (%32.4)	7 (%19.4)	13 (%34.2)	0.31
WBC, mean ± SD, x10^3^/uL	8.35 ± 1.98	8.44 ± 1.82	9.9 ± 2.71	0.01
Hb,mean ± SD, g/dL	14.6 ± 1.7	13.7 ± 1.2	14.1 ± 1.2	0.20
N/Lmean ± SD	1.32 ± 0.3	2.75 ± 0.53	5.78 ± 2.13	0.01
Platelet, mean ± SD, 10^3^/uL	246 ± 55	248 ± 50	236 ± 51	0.61
BUN,mean ± SD, mg/dL	16.9 ± 16.4	16.4 ± 9.8	19 ± 7.4	0.54
Creatinine, mean ± SD, mg/dL	0.89 ± 0.18	0.81 ± 0.17	0.86 ± 0.18	0.15
CK-MB, [IQR] ng/mL	22 [19]	20 [16.2]	24 [19.5]	0.72
Total cholesterol, mean ± SD, mg/dL	191 ± 50	181 ± 36	185 ± 44	0.66
Triglycerides, mean ± SD, mg/dL	170 ± 70	162 ± 70	160 ± 90	0.66
HDL,mean ± SD, mg/dL	41 ± 12	40 ± 8	42 ± 10	0.64
LDL,mean ± SD, mg/dL	116 ± 43	111 ± 34	118 ± 40	0.71
CRP, [IQR] mg/dL	0.5 [0.75]	0.7 [0.75]	1.1 [2.4]	0.01
Troponin I, [IQR] ng/ml	0,02 [0,15]	0,03 [1,1]	0,14 [2,1]	0,05
Recurrent angina, (n%)	9 (%24.3)	13 (%36.1)	26 (%68.4)	0.01
Recurrent MI, (n%)	4 (%10.8)	3 (%8.3)	14 (%36.8)	0.01
Death	0	1	0	--

Abbreviation: BUN, blood urea nitrogen; CK- MB, creatinin kinaz myocardial band; CRP, C-reactive-protein; Hb, hemoglobin ; HDL, high density lipoprotein; LDL, low density lipoprotein; N/L,neutrophil lymphocyte ratio; WBC, white blood cell

**Figure 1:**
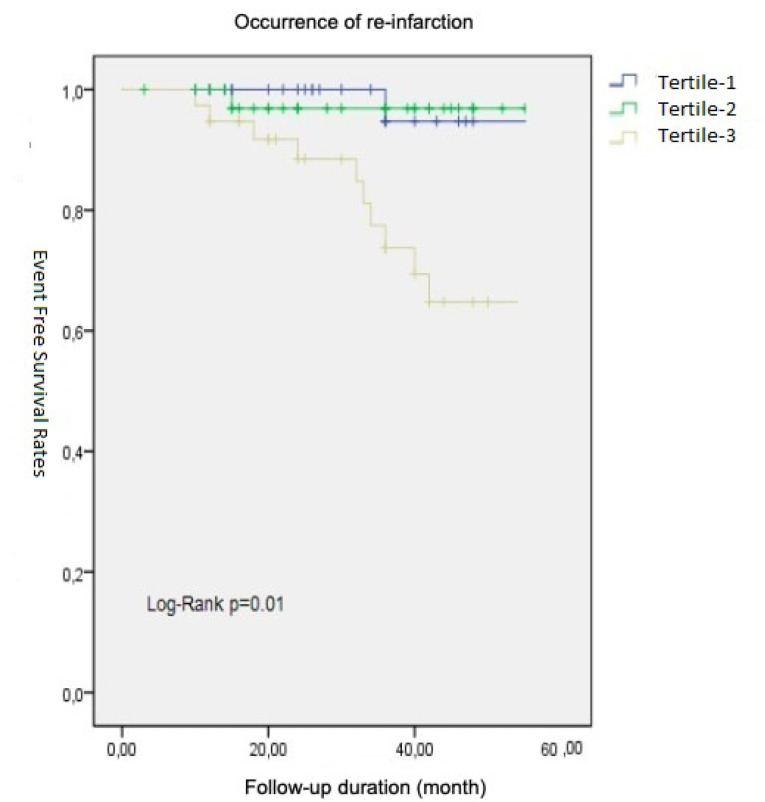
Event free survival rates between different NLR tertiles

The mean follow-up duration was 35.4 ± 1.7 months. In this time period emergency department visits for recurrent angina and hospitalization for recurrent myocardial infarction were considerably high in the highest NLR tertile (%24.3, %36.1, %68.4 *P* < 0.01 and %10.8, %8.3, %36.8 *P* < 0.01 respectively for across tertiles). These data are summarised in Table 1. Long term event (recurrent MI) free survival curves among different tertiles designated by Kaplan-Meier method and compared by the log-rank test depicted in Figure 1.

We performed univariable and multivariable binary logistic regression analyses for all variables in order to find the independent predictors of recurrent myocardial infarction in the long term follow up. Univariate regression analyses showed that smoking, high CRP and neutrophil to lymphocyte ratio levels were correlated with recurrent myocardial infarction in the long term follow up. When we put these variables into the multivariate regression analyses smoking and high NLR levels were ascertained as independent predictors of recurrent myocardial infarction (HR:4.64 95%CI 0.95-22.52 *P*;=0.04, HR: 1.48 95%CI 1.16-1.90 *P* < 0.01 respectively). These data are depicted in Table 2. Owing to the fact that, only one death occurred during the follow up period, we were not able to investigate the relationship between NLR levels and mortality.

**Table 2:** Independent predictors of recurrent myocardial infarction in the long term follow up in multivariate regression analysis

**Variable**	**Adjusted HR (%95 CI)**	***P*** ** value**
CRP	1.04 (0.98-1.11)	0.14
Smoking	4.64 (0.95-22.52)	0.04
NLR	1.48 (1.16-1.90)	0.01

Abbreviations: CRP, C-rective protein; NLR, neutrophil lymphocyte ratio; HR, hazard ratio *NLR values presented as continuous variables.

We had also checked Tolerance and Variance Inflation Factor (VIF) for all parameters included in the regression model in order to prevent multicollinearity. According to the multicollinearity statistic, the tolerance values were >0.1 and VIF values were <10 for all parameters. Therefore, we determined that there was no multicollinearity between each of the variables in the regression model.

## Discussion

The main finding of our study was that, high NLR values on admission were significantly associated with recurrent angina and myocardial infarction in the long-term follow-up of patients presented as NSTE-ACS and had coronary slow flow on angiography.

The exact mechanism of coronary slow flow (CSF) is still unclear. However, it seems that increased microvascular resistance and endothelial dysfunction are the most likely contributing factors. Resting coronary microvascular resistance tend to be higher compared with hyperemia which is objectively displayed with invasive hemodynamic measurements by Fineschi et al.^10^ Endothelin-1 levels, a strong vasoconstrictor mediator, have been demonstrated to be elevated in CSF patients reflecting increased vasomotor tonus.^11^ Impaired endothelial function looks like another pathophysiological mechanism responsible for coronary slow flow as it has been in atherosclerosis. Endothelium dependent vasodilatation mediated by nitric oxide activity has proved to be considerably lower in CSF patients.^12^ Endothelial thickening and luminal narrowing have been showed in pathology specimens taken from coronary slow flow patients’ ventricles.^13,14^ Thus, it has been emphasized that CSF may be a manifestation of subclinical atherosclerosis.^15^ Hence, both CSF and obstructive coronary artery disease (CAD) share common risk factors and clinical manifestations. While the most encountered symptom has been chest pain related to exercise (%51), angina at rest (%34) or dyspnea may be other presentations. Beside these findings, %15 of patients can be recognized with acute coronary syndrome.^3^

Nowadays, we know that atherosclerosis is a complex chronic inflammatory process.^16^ Immune cells, particularly neutrophils and monocytes are actively involved in this course and detected frequently in atherosclerotic plaques. Also, it is believed that these cells can excrete molecules that initiating plaque rupture or progression.^17,18^ Similar evidence has been existed in CSFP. Important mediators of inflammation, such as interlokin-1 and interlokin-6 or serum soluble adhesion molecules like intracellular adhesion molecule-1 (ICAM-1) and vascular cell adhesion molecule-1 (VCAM-1) have been observed significantly higher in CSF patients.^19,20^ Inflammation affects the distribution of immune cells. Furthermore, parameters like neutrophil to lymphocyte ratio, platelet to lymphocyte ratio, mean platelet volume, red cell distribution range can all be acquired from blood samples rapidly without additional costs. Neutrophil to lymphocyte ratio has gained importance in many cardiovascular disorders as well as coronary slow phenomenon. It has been shown to be an independent prognostic indicator in acute ST segment elevation myocardial infarction. In hospital and long-term mortality and the occurrence of heart failure were increased in a study recruiting 2410 participants. ^21^ Stent thrombosis and no-reflow which are associated with substantial morbidity were also increased in patients who have high NLR on admission. ^22,23^ There are huge amount of data indicating that CSF patients have high NLR compared with patients that have normal coronary flow on their angiograms^7,24^ Nevertheless, whether NLR can predict event free survival or mortality in the long-term follow-up at CSF patients remains to be clarified.

Major clinical characteristics of our study population was to be young and active smokers which was consistent with the literature.^24^ The most bothersome part of the clinical course in CSFP patients is recurrent chest pain particularly at rest. Almost eighty percent of patients experience anginal episodes which are frequently require medical attention.^24^ Recurrent angina was observed %43 of patients in our cohort and the frequency reached %68 in the highest NLR tertile which was statistically significant (*P* < 0.01). Nineteen percent of patients experienced recurrent myocardial infarction and re-hospitalized in the follow up period comparable with older studies.^24^ NLR levels and smoking status were independently associated with recurrent myocardial infarction. Beside these, patients in the upper tertile (NLR ≥3.88) had substantially had more myocardial infarction in the long term follow up compared across tertiles. These patients required more emergency department visits and more hospitalization for recurrent myocardial infarction.

This study has some limitations, such as it was a retrospective study with a small sample size. On the other hand we evaluated just one value of NLR rather than consecutive measurements.

## Conclusion

In conclusion, neutrophil to lymphocyte ratio is an inflammatory marker which can be adopted for prognosis in many cardiovascular diseases. High neutrophil to lymphocyte ratio (NLR>3.88) was independently associated with more frequent recurrent myocardial infarction in the long term follow up of NSTE-ACS patients who had coronary slow flow on angiography. Thus, high NLR levels, may aid clinicians for further risk stratification and force for more aggressive therapies.

## Acknowledments

None.

## Competing interest

The author(s) declared no potential conflicts of interest with respect to the research, authorship, and/or publication of this article.

## Ethical approval

This study was approved by University of Health Scienses, Dr. Siyami Ersek Training and Research Hospital ethic committee. (No. 28001928-903.99)

## Funding

The author(s) received no financial support for the research, authorship, and/or publication of this article.
